# Efficacy of Clopidogrel and Clinical Outcome When Clopidogrel Is Coadministered With Atorvastatin and Lansoprazole: A Prospective, Randomized, Controlled Trial

**DOI:** 10.1097/MD.0000000000002262

**Published:** 2015-12-18

**Authors:** Jian-rong Zhang, Di-qing Wang, Jun Du, Guang-su Qu, Jian-lin Du, Song-bai Deng, Ya-jie Liu, Jin-xi Cai, Qiang She

**Affiliations:** From the Department of Cardiology, The Second Affiliated Hospital of Chongqing Medical University, Chongqing, China (J-rZ, D-qW, JD, G-sQ, J-lD, S-bD, Y-jL, QS); Department of Cardiology, The DongNan Hospital, Chongqing, China (J-rZ); and Department of Hematology, The Second Affiliated Hospital of Chongqing Medical University, Chongqing, China (J-xC).

## Abstract

This prospective, randomized, nonblind, controlled trial evaluated the effects of clopidogrel on platelet function upon coadministration with atorvastatin and lansoprazole.

One hundred four adult patients with non-ST-segment elevated acute coronary syndrome (NSTE-ACS) who underwent percutaneous coronary intervention (PCI) with drug-eluting stent implantation were included. All patients were treated with standard dual antiplatelet therapy (DAPT) plus rosuvastatin 10 mg daily after the operation. On the sixth day after PCI, patients were randomly divided into 4 groups, Group A: DAPT + atorvastatin 20 mg daily (a change from rosuvastatin to atorvastatin) + lansoprazole 30 mg daily, Group B: DAPT + atorvastatin 20 mg daily (a change from rosuvastatin to atorvastatin), Group C: DAPT + lansoprazole 30 mg daily (continuing to take rosuvastatin), Group D is the control group. Additional drugs were used according to the situation of patients. Platelet function and concentrations of platelet activation markers (granular membrane protein 140 (P-selectin), thromboxane B2 (TXB2), and human soluble cluster of differentiation 40 ligand (sCD40L)) were assessed before randomization and at 15- and 30-day follow-up visits. All patients were maintained on treatment for 6 months and observed for bleeding and ischemic events.

A total of 104 patients were enrolled, 27 patients in group A, 26 patients in Group B/C, 25 patients in Group D separately, and all the patients were analyzed. There were no differences in platelet function and the levels of platelet activation markers (P-selectin, TXB_2_, and sCD40L) among or within the 4 groups at the 3 time points of interest (*P* > 0.05). In the subsequent 6 months, no significant bleeding events occurred, and 12 patients experienced ischemic events, these results were also not significantly different among the groups (*P* > 0.05).

In patients diagnosed with NSTE-ACS who have had drug-eluting stent implantation, simultaneously administering clopidogrel, atorvastatin, and lansoprazole did not decrease the antiplatelet efficacy of clopidogrel or increase adverse event frequency over 6 months.

## INTRODUCTION

Clopidogrel is an important antiplatelet drug that is widely used to prevent vessel blockage in clinical settings such as cardiovascular and cerebrovascular diseases. Dual antiplatelet therapy with clopidogrel and aspirin has become the standard treatment for acute coronary syndrome and after percutaneous coronary intervention (PCI).^1^ Additionally, clopidogrel is commonly used with statins to lower the blood lipid level and with proton pump inhibitors (PPIs) to counteract gastrointestinal tract disturbances such as aspirin-induced bleeding.

The effects of clopidogrel on platelets vary among patients, with approximately 4% to 30% of patients being low responders or nonresponders^2^ and having an increased risk of ischemic events after stent implantation.^3–5^ The interaction between clopidogrel and other drugs may promote ischemic events, as evidenced by emerging data that clopidogrel's effect on platelet function is altered by coadministration with statins or PPIs.

CYP3A4 and CYP2C19 are the most important isozymes of cytochromeP450 (CYP450), which activates clopidogrel. Fat-soluble statins are mainly metabolized by CYP3A4, and most PPIs are metabolized by CYP2C19. When clopidogrel is coadministered with fat-soluble statins or PPIs, a drug interaction may occur because of binding site competition. Our study is a prospective, randomized, controlled trial that assesses platelet function and the platelet activation index in plasma to evaluate drug interactions when clopidogrel is simultaneously coadministered with fat-soluble statins and PPIs, providing a reference for clinical practice.

## METHODS

### Patients

All patients were 18 years or older, diagnosed with non-ST-segment elevated acute coronary syndrome (NSTE-ACS) and had undergone PCI in the present study. NSTE-ACS includes unstable angina and non-ST-segment elevation myocardial infarction. According to clinical guidelines, unstable angina refers to the following situations: resting state angina, with a duration often greater than 20 minutes; newly discovered angina within 1 month; angina deterioration within 1 month, with more frequent seizures, more serious pain, or longer pain duration; variant angina pectoris; and angina attack causing electrocardiogram performance for at least 2 adjacent ST segments to decrease ≥0.1 mV or transient ST-segment elevation. Non-ST-segment elevation myocardial infarction is angina with increased myocardial injury markers in the blood. The exclusion criteria were as follows: angina pectoris after infarction; use of clopidogrel/PPIs/statins within the past 2 weeks; known use of CYP3A4 or CYP2C19 inhibitors or activators, such as ketoconazole, rifampin, or erythrocin; use of a glycoprotein IIb/IIIa receptor inhibitor, warfarin, or cilostazol in the perioperative period; a high risk of gastrointestinal bleeding; ALT and AST ≥3 times the normal; renal insufficiency (Cre < 25 mL/min); a platelet count < 100 × 10^9^/L or >300 × 10^9^/L; and expected survival time less than 1 year because of nonheart disease. The following general information was collected: age, gender, BMI, smoking history, vessel stent implantation, chronic diseases, drugs, ALT, AST, PLT, BMI, LVEF, TG, TC, LDL-C, and HDL-C.

The study protocol was approved by the Ethics Committee of Chongqing Medical University and the scientific research department of our hospital. All experimental procedures were performed in accordance with the principles of the Helsinki Declaration. Each patient provided written informed consent.

### Study Design

**FIGURE 1:**
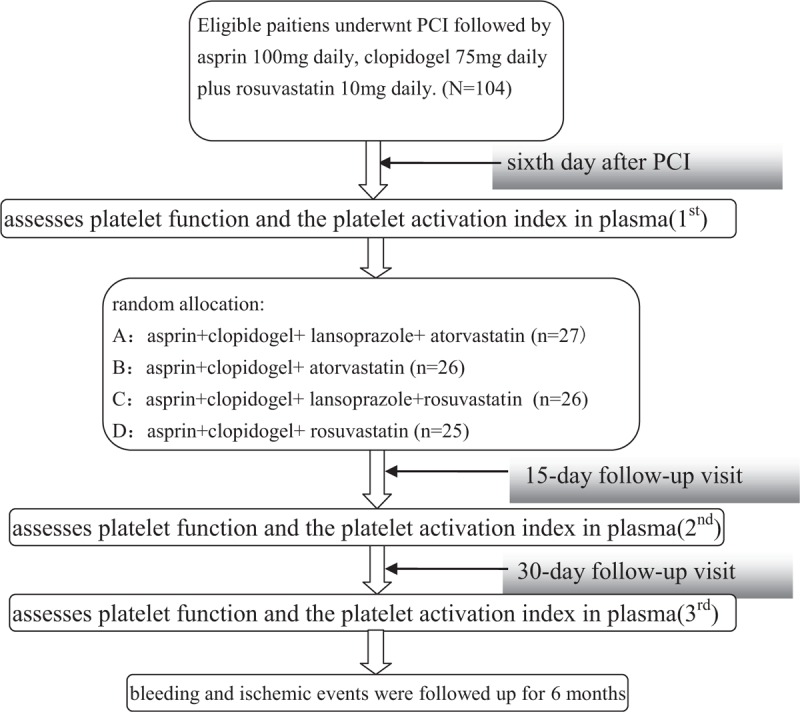
Flowchart of the study.

All eligible participants were treated with aspirin 300 mg and clopidogrel 300 mg for the loading dose (except for long-term aspirin users) 6 to 12 hours before PCI, followed by standard dual antiplatelet therapy (DAPT) (aspirin 100 mg daily and clopidogrel 75 mg daily) after surgery. Rosuvastatin 10 mg daily was used for antiinflammation therapy. Other drugs, such as ACEI, ARB, β-blockers, and oral hypoglycemic drugs, were used as usual, according to the needs of the patients. The inhibition of platelet aggregation rate (IPA%, stimulation with 2 μmol/L adenosine diphosphate (ADP) or 1 mmol/L arachidonic acid (AA) 10 μL, respectively) and the ADP-induced maximum clot strength (MA_ADP_) were assessed by thrombelastogram (TEG), and the concentration of P-selectin, TXB_2_, sCD40L in plasma were assessed by ELISA on the sixth day after PCI (first). After the first assessment, patients were randomly divided into 4 groups by random number table method: Group A: DAPT + atorvastatin 20 mg daily (a change from rosuvastatin to atorvastatin) + lansoprazole 30 mg daily, Group B: DAPT + atorvastatin 20 mg daily (a change from rosuvastatin to atorvastatin), Group C: DAPT + lansoprazole 30 mg daily (continuing to take rosuvastatin), Group D is the control group. Then, we retested the index 15 and 30 days after randomization (second and third). All patients were maintained on treatment for 6 months and observed for bleeding and ischemic events (Fig. 1). According to the measurement of TIMI standard, minor bleeding refers to clinically significant bleeding with a fall in hemoglobin of 3.0 to 5.0 g/dL or a fall in hematocrit of 9% to <15%. Major bleeding refers to hemoglobin >5 g/dL or a hematocrit decease ≥15%.^6^ Ischemic events include death, stroke, myocardial infarction (in-patient or after discharge), rehospitalization due to angina, and cardiovascular revascularization.

The index was measured via ELISA according to the manufacturer's instructions. The ELISA kits all were purchased from WuHan ColorfulGene Biological Technology Co., Ltd (China). Catalog numbers: Human P-selectin ELISA kit DRE10447, Human soluble cluster of differentiation 40 ligand (sCD40L) ELISA kit DRE10444, Human thromboxane B2 (TXB2) ELISA kit DRE10984.

### Platelet Function Test

TEG was used to quantitatively analyze platelet function by testing fresh whole blood thrombin-induced clot strength. Gurbel et al compared TEG and light transmittance aggregometry (LTA) on the prognosis of patients with platelet function testing results and their correlation, and they showed that TEG is significantly better than LTA for predicting long-term events. TEG can be used as a measuring method to guide individualized antiplatelet therapy.^3,7^ Stimulation with 2 μmol/L ADP or 1 mmol/L AA 10 μL leads to platelet aggregation, which can detect the P2Y12 or cyclooxygenase pathways to determine the impact on blood clot formation. The IPA% in response to ADP or AA is calculated with computerized software based on the following formula:  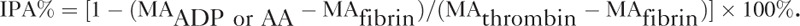. MA_ADP_ between 31 and 47 mm is considered the clopidogrel treatment safety margin, where <31 mm increases the risk of hemorrhage, and >47 mm increases the risk of thrombosis.^7^

### Platelet Activation Markers

Activated TXB is an AA metabolite, whose precursor is TXA, and its synthesis begins on platelet membrane phospholipids. Stimulated by a thrombus, TXA_2_ is generated, which is subsequently converted into stable metabolites TXB_2_. Therefore, determining the TXB_2_ levels can reflect TXA_2_ levels and indirectly assess platelet activation status.

P-selectin (also called CD62p or GMP-140) is a glycoprotein within Weibel–Palade bodies, which are located in blood platelets and endothelial cells. P-selectin is a specific molecular marker for platelet activation. When platelets are activated, the P-selectin concentrations on the platelet membranes and in plasma are increased.

When platelets are activated, the expression of the platelet membrane marker CD40L increases. The marker can enter the circulation and form soluble CD40L (sCD40L), which is the activated form of CD40L. sCD40L can combine with CD40-activated platelets to induce platelets to release the α particle and dense granules, thus increasing the expression of P-selectin. Thus, increased plasma-soluble CD40L is also a sign of platelet activation.

### Statistical Analysis

SPSS statistical software was used. Count data are expressed as frequencies and percentages and were analyzed by the Pearson χ^2^ test or the Fisher exact test. Continuous measurement data are expressed as X ± S. Stratified according to time, comparisons among multiple groups were performed with single factor analysis of variance, and comparisons between 2 groups were performed with the SNK test. Stratified according to group, comparisons among multiple detecting points were performed with repeated measures analysis of variance, and comparisons between 2 detecting points were performed with the Bonferroni test. Kaplan–Meier survival curves were used to compare the incident-free survival rate among groups. *P* < 0.05 was considered significant.

## RESULTS

### General Clinical Data

In total, 104 consecutive eligible patients were chosen for the study, including 81 males and 23 females. The average age is 63 ± 9 years old. All patients were successfully completed the experimental process. The baseline clinical characteristics were not different (*P* > 0.05) (Table 1).

**TABLE 1:**
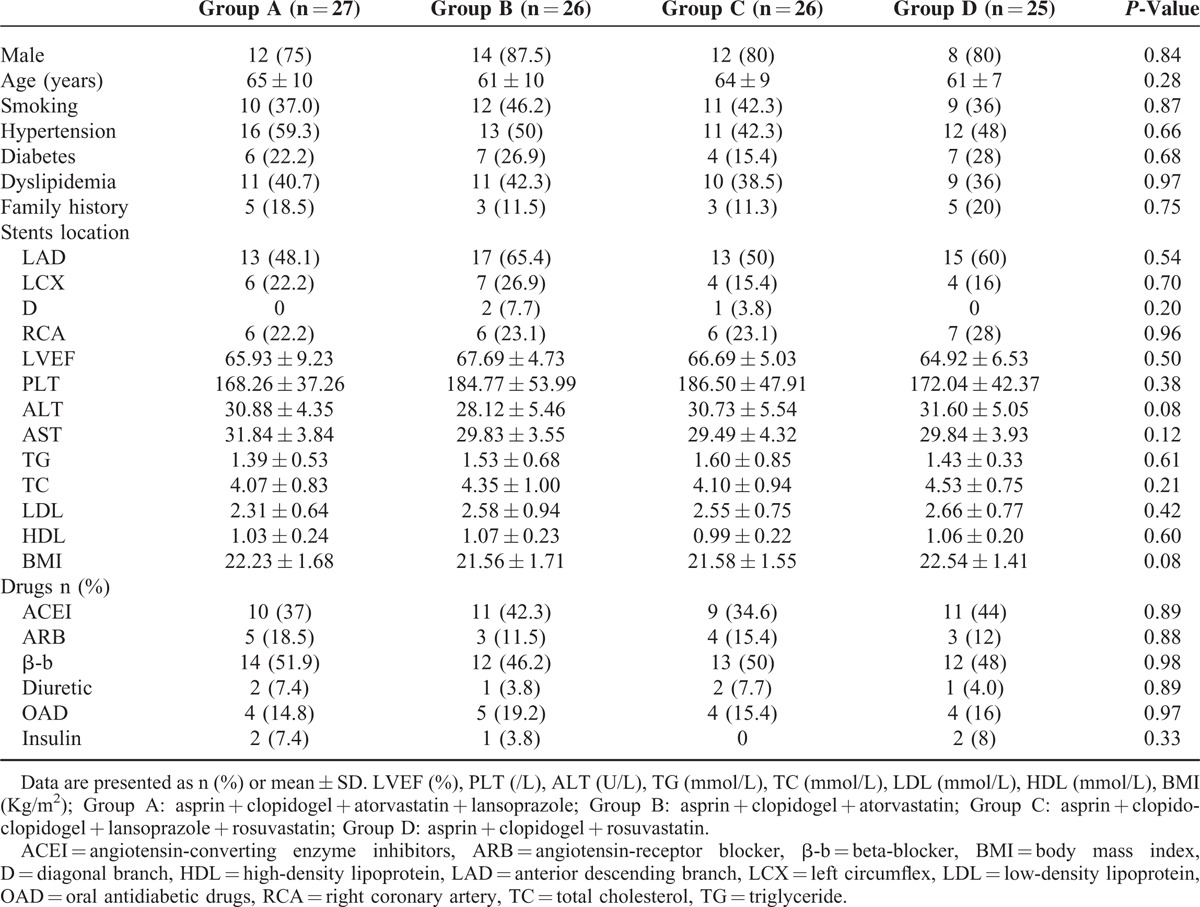
Baseline Characteristics of the Study population

### Comparison Among Groups

Stratified according to time, the results at first to third were compared. The ADP-IPA%, AA-IPA%, MA_ADP_, P-selectin, sCD40L, TXB_2_ results showed no significant differences among groups (*P* > 0.05). Thus, when clopidogrel is coadministered with atorvastatin or lansoprazole, these drugs do not influence platelet function or platelet activation (Table 2).

**TABLE 2:**
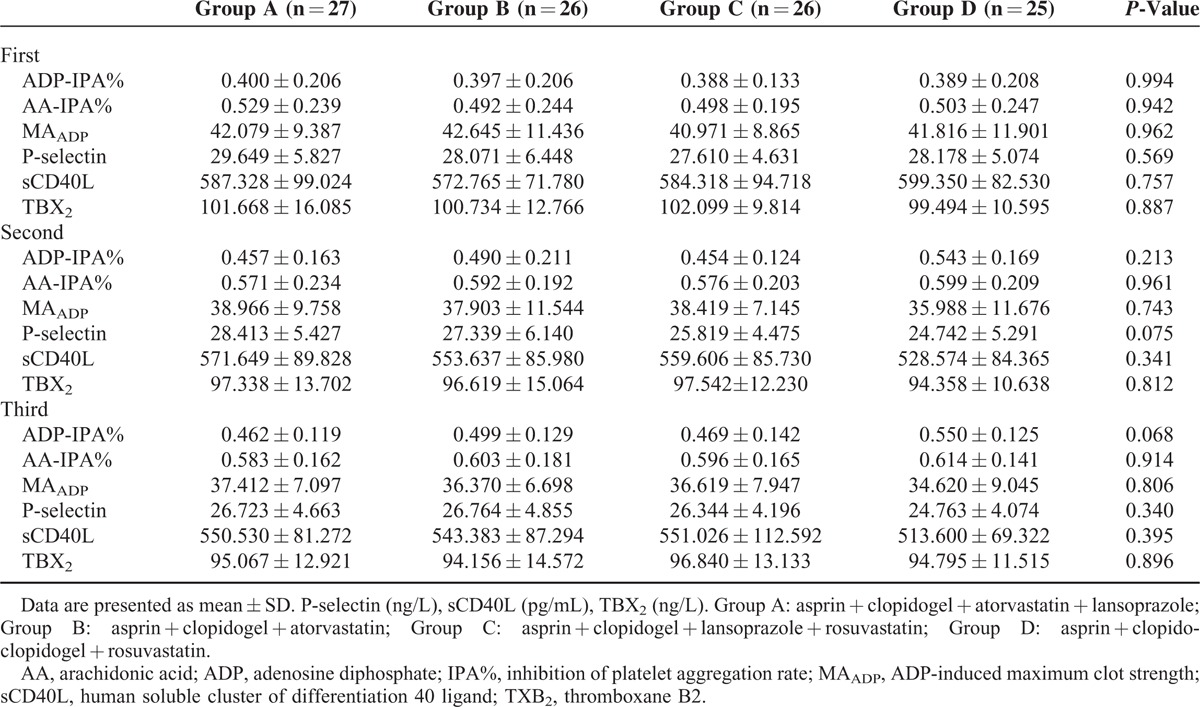
Comparison of Platelet Function and Concentrations of Platelet Activation Markers Among Groups

### Intragroup Comparison

Stratified according to group, the results in each group were compared. ADP-IPA% showed an increasing trend in all groups, while MA_ADP_, P-selectin, sCD40L, and TXB_2_ all showed gradually declining trends. The intragroup comparison showed no difference in Groups A, B, and C, as was the case for comparisons between any 2 results. However, in Group D, these trends are more obvious than in the other groups. The differences among the results of ADP-IPA%, MA_ADP_, P-selectin, and sCD40L at each time point are significant (*P* < 0.05, respectively), which indicates that the platelet aggregation induced by ADP and platelet activation decreased. Thus, the patients’ responses to clopidogrel were enhanced over time. Comparison between any 2 results indicated that the changes in trends led to significant differences after taking clopidogrel for 15 days. Additionally, the result of taking clopidogrel for 30 days was not significantly different compared with 15 days. There were no significant differences among the rest of the indicators in group D (Table 3).

**TABLE 3:**
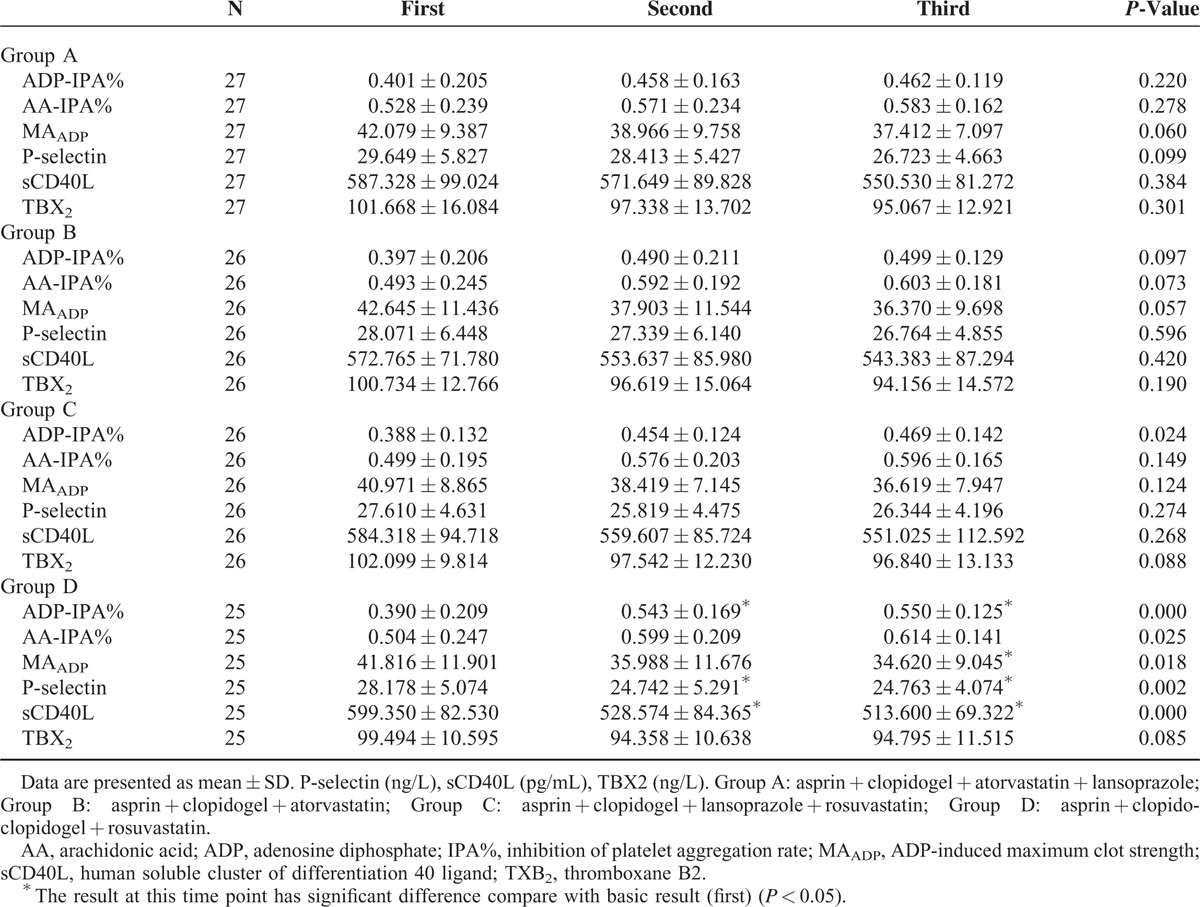
Comparison of Platelet Function and Concentrations of Platelet Activation Markers From the First to Third Time Points in Each Group

### Clinical Outcome

Bleeding and ischemic events were followed up for 6 months, and all patients did not experience bleeding events. Twelve people experienced ischemic events, including 7 people with angina pectoris readmission, 4 people with cardiovascular revascularization, and 1 person with myocardial infarction. There were no significant differences among the groups (*P* = 0.95) (Table 4).

**TABLE 4:**
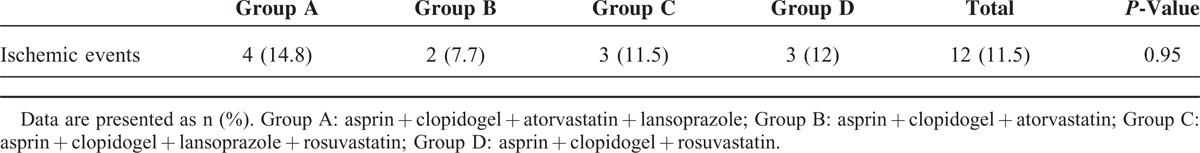
The Occurrence of Ischemic Events

**FIGURE 2:**
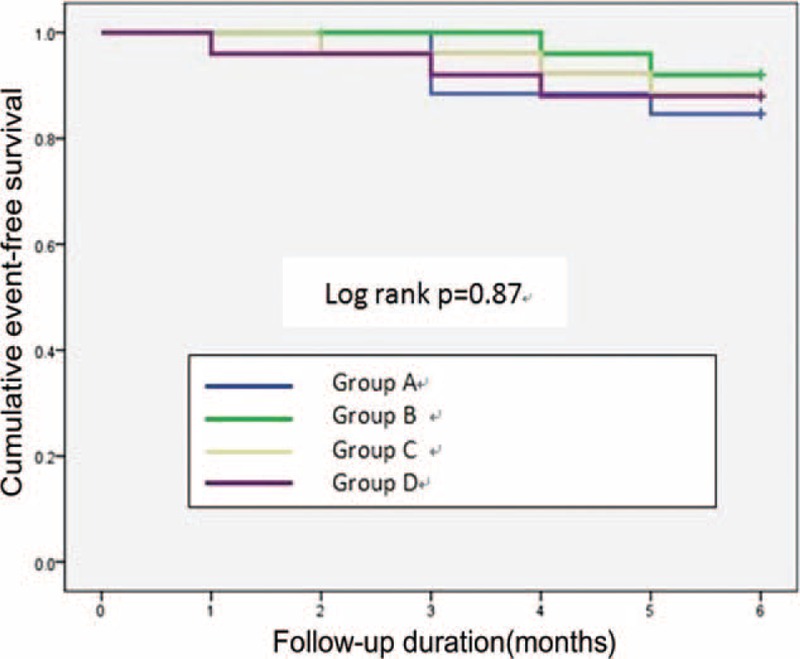
Comparison of the cumulative event-free survival rates among groups. Group A: asprin + clopidogel + atorvastatin + lansoprazole; Group B: asprin + clopidogel + atorvastatin; Group C: asprin + clopidogel + lansoprazole + rosuvastatin; Group D: asprin + clopidogel + rosuvastatin.

The Kaplan–Meier survival curve analysis results indicated that the ischemic event-free survival rate was not significantly different among the groups within 6 months after PCI (*P* = 0.87) (Fig. 2).

## DISCUSSION

In this study, in patients with NSTE-ACS and a drug-eluting stent, who received conventional dual antiplatelet combined with atorvastatin, the platelet aggregation inhibition rate and platelet activation did not change within 1 month, and this treatment did not increase bleeding or ischemic events compared with cotreatment with rosuvastatin, which is not metabolized by CYP3A4. Fat-soluble statins (eg, atorvastatin, lovastatin, simvastatin) are mainly metabolized by the CYP450 isoenzyme CYP3A4, which should activate clopidogrel. When these drugs are used in combination, they may interact because of binding site competition and this coadministration may inhibit clopidogrel activation and reduce the antiplatelet effect, but these results have been controversial until now. In 2003, Lau et al^8^ reported that atorvastatin can decrease the activation of clopidogrel by 90% and reduce the antiplatelet effect of clopidogrel in vitro experiments, which have drawn wide attention. Pravastatin and rosuvastatin are water-soluble statins that are not metabolized through CYP3A4, and they do not influence clopidogrel activity.^8–13^ Park et al^14^ reported that replacing a fat-soluble statin with the water-soluble statins pravastatin or rosuvastatin in patients with long-term use of clopidogrel and atorvastatin (the ACCEL-STATIN study) results in decreasing platelet activity. Brophy et al^15^ and Gulec et al's^16^ observational studies found that clopidogrel will increase cardiac adverse events in patients with coronary artery stent implantation when it was coadministered with atorvastatin or simvastatin compared with rosuvastatin or pravastatin. Furthermore, multiple studies have found no interaction between atorvastatin or simvastatin and clopidogrel,^17–20^ and denied that coadministering CYP3A4-metabolized statins with clopidogrel can increase the frequency of clinical adverse events.^9,21–23^

In our study, the use of clopidogrel in combination with lansoprazole (Group C) compared with the no-PPIs group (Group D) did not reduce the clopidogrel platelet inhibition rate and did not increase the degree of platelet activation, and 6-month clinical endpoint events were also not significantly different. Most PPIs (eg, omeprazole, lansoprazole, pantoprazole, rabeprazole, esomeprazole) are metabolized by the CYP450 isozyme CYP2C19, which is also an important metabolic enzyme that participates in clopidogrel activation. The possible drug interactions between PPIs and clopidogrel have been extensively researched. Thus far, the most studied PPI is omeprazole, which has an inhibitory effect on clopidogrel in several studies.^24–26^ In 2008, the United States Food and Drug Administration (FDA) warned of the dangers of combining clopidogrel and omeprazole.^27^ Indeed, Li et al^28^ compared the commonly used PPIs (omeprazole, lansoprazole, rabeprazole, pantoprazole, and esomeprazole) for competitive inhibition intensity of CYP2C19 and found that the competitive inhibition intensity of lansoprazole is the highest, whereas that of pantoprazole is the lowest. Burkard et al^29^ observed the adverse events of patients who received PCI and underwent 6 months of clopidogrel and PPI therapy (the BASKET trial). The results show that the combined use of PPIs may increase the risk of acute myocardial infarction in the following 36 months and that such use is an independent predictor of long-time clinical outcome. Unlike these results, other researches^30,31^ suggest that clopidogrel and PPIs are safe when used in combination. In a word, this problem requires further study.

In our study, clopidogrel coadministered with atorvastatin and lansoprazole (Group A) simultaneously did not reduce the platelet inhibition of clopidogrel and did not increase platelet activity within 1 month after PCI, and postoperative bleeding and ischemic event frequencies after 6 months showed no differences compared with other experimental groups and the control group. Thus, in patients with drug-eluting stent implantation, simultaneously using clopidogrel, atorvastatin, and lansoprazole is safe. Drug interactions are influenced by many factors. Clopidogrel is metabolized through CYP450, a variety of isozymes are involved in this process, such as CYP1A2, CYP2C9, CYP2D6, and CYP3A5,^32–35^ except the 2 most important CYP3A4 and CYP2C19. The combination of CYP3A4 and CYP2C19 inhibitors may be not sufficient to affect the metabolism of clopidogrel. Furthermore, the clinically common fat-soluble statin and PPI drug doses might not reach the saturation concentration of CYP3A4 and CYP2C19, thereby not producing competitive inhibition, which may partially explain our results.

We also observed that for clopidogrel in combination with rosuvastatin (Group D), ADP-IPA% shows an increasing trend, whereas MA_ADP_, P-selectin, sCD40L, and TBX_2_ showed gradually declining trends. Additionally, the results at each time point assessed were significantly different. That suggest the antiplatelet effect of clopidogrel increased over time, which is consistent with the result of Campo et al’ study.^36^ Comparison between any 2 time points indicates that there was a significant difference after taking clopidogrel for 15 days. These differences were observed but not significant for Group A/B/C, therefore, we speculate that there may be drug interactions between clopidogrel, atorvastatin and lansoprazole, and the gradual increase in the reactivity of clopidogrel may be weakened because of the interaction between these drugs, resulting in no difference in final performance. However, platelet activation and platelet inhibition rate did not change significantly over time for Group A/B/C, which suggests that the influence of atorvastatin or lansoprazole on clopidogrel efficacy is extremely weak and will not affect clinical events.

The investigation suffers some limitations. First, the study sample size is small. Second, in our study, atorvastatin and lansoprazole were only used at the conventional treatment doses, so any drug interaction between high doses of these drugs and clopidogrel remain unclear. Lastly, we did not test platelet function and the concentration of platelet activation markers at 6 months post-PCI.

In conclusion, our study suggest patients diagnosed with NSTE-ACS who underwent PCI with drug-eluting stent implantation and who were simultaneously administered clopidogrel, atorvastatin, and lansoprazole, the antiplatelet efficacy of clopidogrel did not decrease, and adverse events did not increase over 6 months.
